# Highly Flexible Methyl Cellulose/Gelatin Hydrogels for Potential Cartilage Tissue Engineering Applications

**DOI:** 10.1002/bip.23641

**Published:** 2025-01-07

**Authors:** Mehmet Ali Karaca, Vida Khalili, Duygu Ege

**Affiliations:** ^1^ Institute of Biomedical Engineering Boğaziçi University Istanbul Turkey; ^2^ Institut für Werkstoffe Ruhr‐Universität Bochum Bochum Germany

**Keywords:** cartilage, gelatin, mesenchymal stem cells, methyl cellulose, NHS/EDC

## Abstract

Cartilage damage resulting from trauma demonstrates a poor capacity for repair due to its avascular nature. Cartilage tissue engineering offers a unique therapeutic option for cartilage recovery. In this study, methylcellulose (MC)/gelatin (GEL) hydrogels (MC10G20, MC12.5G20, MC15G20, and MC17.5G20) were developed to assess and compare their chemical, mechanical, and biological characteristics for cartilage repair. First, the interaction between MC and GEL after blending and subsequent crosslinking with EDC/NHS was confirmed by using FTIR. Mechanical tests under compression test revealed that hydrogels' resistance to both elastic and plastic deformation increased with higher wt.% of MC. The % strain of the hydrogels doubled with the addition of MC, likely due to abundant hydrogen bonding between polymeric chains. Furthermore, the compressive modulus of MC/GEL hydrogels was approximately 0.2 MPa, closely matching modulus of human cartilage tissue. Similarly, the % water retention capacity of the hydrogels increased over the 7 days as the MC content increased. Additionally, SEM images showed that the incorporation of MC to GEL introduced porosity with the diameters ranging from 10 to 50 μm, similar to the size of pores in native cartilage. In vitro cell culture studies confirmed the biocompatibility of MC/GEL hydrogels. Fluorescence staining showed a 2.5‐fold increase in F‐actin staining following the incorporation of MC into the hydrogels. Overall, this study highlights the potential of MC/GEL hydrogels for cartilage tissue engineering, however, further research is required to assess its full potential.

## Graphical abstract

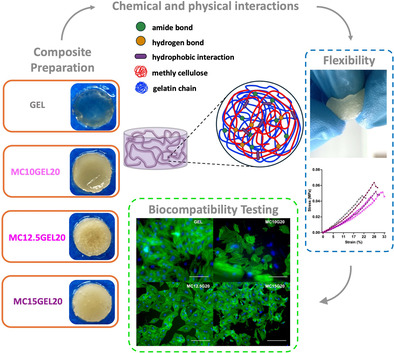

## 1 Introduction

Cartilage, with its limited regenerative capacity, presents a significant challenge when damaged by injury and degenerative conditions [1]. The intrinsic inability of cartilage to efficiently self‐repair shows the importance of exploring alternative strategies for restoration [2, 3, 4]. Tissue engineering has emerged as a promising field, utilizing biomaterials to support the cartilage regeneration [5, 6, 7, 8].

Methyl cellulose (MC) is a water‐soluble natural carbohydrate exhibiting unique thermo‐reversible gelation properties [9]. Derived from cellulose by etherification reaction, MC is a promising candidate in tissue engineering applications. However, it lacks appropriate cell culture conditions for mammalian cells due to the absence of cell attachment sites such as integrin‐binding sites [10].

Gelatin (GEL), derived from collagen, is a biocompatible and biodegradable biomaterial with inherent gelation capabilities [11]. As a natural protein, GEL can undergo gelation just above ambient temperatures, making it an ideal biomaterial in tissue engineering [12]. Additionally, GEL contains RGD peptide sequence, a principal binding domain found in ECM proteins, which promotes cell attachment to biomaterial scaffolds [13, 14].

Literature indicates that MC and RGD motif containing polymeric hydrogels provide suitable 3D porous scaffolds for tissue engineering applications [15, 16, 17]. In the study of Rastin et al. [16], the addition of 8 wt.% of MC to 5 wt.% of GelMA hydrogels resulted in high shape fidelity and improved the printability and viscosity [16]. The incorporation of GelMA in the MC bioink enhanced the yield strength as well as storage and loss modulus due to hydrogen bonding between GelMA and MC [16]. Cofiño et al. [17] confirmed that blend formulation of a self‐assembled peptide (RAD16‐I 2.7 wt.%) with 1.5 wt.% of MC (MC in 4× phosphate buffer saline [PBS]) exhibited higher gel strength, and shape fidelity compared to the peptide hydrogel alone [17, 18].

Increasing the cross‐linking density of GEL in hydrogels enhances physical and biological properties of the scaffolds [15, 19]. The Young's modulus of MC‐based hydrogels (composed of 2 wt.% MC, 0.2 wt.% GEL, 0.2 wt.% agarose) increased with cross‐linking density of gelatin by addition of crosslinking agent (carbonyldiimidazole, CDI). This increase also enhanced ALP activity and osteocalcin secretion on Day 14, as well as the expression levels of ALP and OCN genes of hMSCs on Day 15 [15]. Additionally, literature indicates that the chemical crosslinking of gelatin using EDC (1‐Ethyl‐3‐(3‐dimethylaminopropyl)carbodiimide)/NHS (*N*‐hydroxysuccinimide) improved the mechanical performance of hydrogel and may also promote the differentiation of chondrocytes [19, 20].

Given the benefits of MC in enhancing the rheological properties and mechanical performance of gelatin‐based hydrogels, this study presents, for the first time, the preparation of MC/GEL hydrogels with varying wt.% of MC. Gelatin was further crosslinked in the hydrogel using EDC/NHS. Then, their chemical structure, mechanical performance, and degradation behavior are analyzed, and cell culture studies are carried out. These findings show the promising potential of these hydrogels for cartilage tissue engineering. In the future, the outcomes of this study may facilitate the development of 3D‐printed MC/GEL scaffolds for tissue engineering applications.

## 2 Materials and Methods

### 2.1 Preparation of the MC/GEL Hydrogel

The composition of MC/GEL hydrogel samples is present in Table 1. A 20 wt.% solution of GEL (from bovine skin, Type B, Sigma Aldrich) was prepared in distilled water solution and stirred at 50°C for 20 min. Gradually, methylcellulose (15 cP, Sigma Aldrich) powder was added to GEL solutions at the concentration of 10, 12.5, 15, and 17.5 wt.%. The polymer solutions were mixed until the MC was completely dissolved, resulting in MC/GEL solutions. These aqueous solutions were then poured into cylindric Teflon mold columns, allowed to cool to room temperature, and cast into chewing‐gum‐like MC/GEL hydrogel tablets. Finally, the MC/GEL hydrogel tablets were incubated overnight in EDC (N‐(3‐Dimethylaminopropyl)‐*N*′‐ethyl carbodiimide hydrochloride, Sigma Aldrich) solution, which was used as a cross‐linker.

**TABLE 1:** Composition of prepared MC/GEL hydrogel samples.

Abbreviation	Methlycellulose (MC) (wt.%)	GEL (wt.%)
GEL	0	20
MC10G20	10	20
MC12.5G20	12.5	20
MC15G20	15	20
MC17.5G20	17.5	20

### 2.2 Characterization of Methylcellulose–GEL Hydrogel

#### 2.2.1 Fourier Transform Infrared (FTIR)

The vibration state of functional groups of MC/GEL hydrogels was confirmed by using a FTIR spectroscopy (Thermo Scientific Nicolet 380 FTIR Spectrometer) over the range of 4000–400 cm^−1^. FTIR spectrum of each sample was analyzed, and peaks were labeled by OMNIC software. The chemical interaction, crosslinks and functional groups of the MC/GEL hydrogels were compared with those of pure MC and GEL, as well as with finding from previous research.

#### 2.2.2 Mechanical Testing (Compressive Strength Measurement)

The size of MC/GEL hydrogel tablets was measured before the mechanical analyses of the samples. The compression modulus and strength of MC/GEL hydrogel tablets (*n* = 5) were measured using Lloyd single column universal testing machine (LF Plus, Ametek Inc.) under a loading rate of 5 mm/min. The breaking point, as well as load/displacement data for the MC/GEL hydrogel tablets was recorded, and mechanical tests were controlled by software (NEXYGEN Plus). Compressive strength and modulus were calculated from the stress/strain curve. The measurement of the compressive strength, compressive modulus, stress, and % strain data was analyzed using GraphPad Prism.

#### 2.2.3 In Vitro Biodegradation and Protein Release

MC/GEL hydrogel tablets (*n* = 3) were immersed in 5 mL PBS solution and incubated at 37°C. Tablets were photographed at each time points (0, 1, 2, 3, 4, 5, and 7 days). Three images from each group were selected to determine surface area of the samples. The weight (*W*_i_) of the MC/GEL hydrogel tablets after immersion in PBS solution was compared with the weight (*W*_d_) of dried MC/GEL hydrogel tablets. Image analyses were performed using ImageJ software. The in vitro biodegradation of tablets was determined as follows:

$$\text{Weight loss}\%=\left(\left(W_{i}-W_{d}\right)/W_{i}\right)\times 100$$

MC/GEL hydrogel tablets (*n* = 3) were immersed in 5 mL PBS solution and incubated at 37°C. PBS sample were collected each time points (30, 60, 180, 360, 720 min, 1, 3, 5 and 7 days). Total protein concentrations in collected PBS was measured at 280 nm by NanoDrop 2000/2000c (Thermo Scientific). The cumulative amount of GEL (*G*_r_) in PBS was compared with the initial amount (*W*_d_) of GEL in MC/GEL hydrogel tablets [21]. The released GEL from tablets was determined as follows:

$$\mathrm{GEL}\;\text{release}\%=\left(G_{r}/G_{i}\right)\times 100$$

Weight loss (%) and cumulative GEL release (%) were plotted using GraphPad Prism.

#### 2.2.4 Swelling Test and pH Changes

For the swelling study, MC/GEL hydrogel tablets (*n* = 3) were immersed in 5 mL PBS solution and incubated at 37°C. The dry weight (*W*_i_) of the MC/GEL hydrogel tablets were compared with the wet weight (*W*_s_) of MC/GEL hydrogel tablets after immersion for 30, 60, 180 min, as well as 1, 3, 5, and 7 days. The water uptake of tablets was determined as follows:

$$\text{Swelling}\%=\left(\left(W_{s}-W_{i}\right)/W_{i}\right)\times 100$$

MC/GEL hydrogel tablets (*n* = 5) were immersed in PBS solution and incubated at 37°C. The pH of the PBS solutions was measured at various time intervals (60, 180 min, as well as 1, 3, 5 and 7 days) using a pH meter (Thermo Scientific‐Orion Star A2111). The pH values were analyzed using GraphPad Prism.

### 2.3 Cell Culture Studies

Bone marrow derived‐mesenchymal stem cells (BM‐MSCs, ATCC PCS‐500‐012) were cultured in MEM Alpha (Biowest) supplemented with 10% fetal bovine serum (FBS, Biowest), 1% penicillin–streptomycin (PSA, Biowest), 4 mM l‐glutamine (Biowest) at 37°C in a humidified atmosphere 5% CO_2_. The media were replaced with fresh media every 2 days during the 5‐day incubation.

BM‐MSCs were used to assess the biological properties of MC/GEL scaffold. Scaffolds with 1.5 cm in diameter and 1 mm in height were prepared for in vitro cell culture studies and sterilized by UV treatment for 45 min for each side. The sterilized samples were soaked in the medium for 2 h. before cell seeding. Cells at a concentration of 10 × 10^4^ cell/well in 50 μL medium were cultured on the top surface of scaffolds. The scaffolds were incubated for 2 h in an incubator to allow cell attachment to scaffold surface. Then, each well was filled with 450 μL completed medium. Fresh medium was exchanged every 2 days during the 5‐day incubation period. The stability of the scaffold and cells was monitored throughout the incubation of cells.

GEL, MC10G20, MC12.5G20, and MC15G20 samples were examined by using SEM (Thermo Fisher Scientific QuattroS) under high vacuum at 10 kV. Cell‐seeded samples with 1.5 cm in diameter and 1 mm in height were incubated under in vitro cell culture conditions for 5 days and then dried at room temperature. After drying, samples were coated with 3.5 nm of gold–palladium prior to microscopic analysis.

Pore size in the MC10G20, MC12.5G20, and MC15G20 samples were measured using ImageJ software. The pore size values were graphed and analyzed using GraphPad Prism.

Three SEM images of each sample were selected to determine pores area for each group using ImageJ software [22]. The algorithm in ImageJ software uses a histogram‐derived thresholding technique to detect the lowest and highest intensity from two distinct peaks in the histogram of SEM images, representing bright and dark part of image. The black areas in the image represent the pores within the hydrogel material. The pore areas in these black regions are measured for the hydrogel sample using “Analyze Particles” tool in ImageJ software. Pore area % of each sample were determined. Pore area % were graphed and analyzed using GraphPad Prism.

#### 2.3.1 Metabolic Activity and Morphology of BM‐MSCs on MC/GEL Scaffolds

Metabolic activity of BM‐MSCs on the scaffolds was investigated using Alamar Blue assay (Invitrogen, DAL1025) after 3 and 5 days of the cell seeding on the hydrogel mixture, according to the manufacturer's instruction. In this study, the mitochondrial activity of the cells on the hydrogel was evaluated by measuring reduction of resazurin by living cells. The waste medium in the cell culture was replaced with fresh medium containing 10% Alamar blue and the samples were incubated for 4 h at 37°C in a humidified atmosphere with 5% CO_2_. The cell viability was assessed in triplicate wells. The viability % of control well was considered 100% and used as a reference for comparison with experimental groups. The viability % were analyzed using GraphPad Prism.

Morphological assessment of the BM‐MSCs on the hydrogels was conducted using staining of cells with phalloidin‐iFluor 488 reagent for actin filament staining and DAPI for nucleus staining. The cells attached on the plate surface after 5 days were washed with PBS solution and then fixed with 4% paraformaldehyde solution (PFA) for 15 min. Following the fixation, the samples were washed with PBS solution three times and incubated in 0.1% Triton‐X solution. Samples were incubated in 1% BSA blocking buffer solution for 1 h to prevent nonspecific binding. After blocking, the cells were washed with PBS solution three times and stained with phalloidin‐iFluor 488 reagent (Abcam, ab176753) for 1 h. Following the phalloidin‐iFluor 488 staining, the cells were washed with PBS solution for three times and stained with DAPI solution for 30 min. After another round of washing with PBS, the BM‐MSCs were visualized using fluorescence microscope (Zeiss Axio Vert.A1 inverted microscope for advanced routine). Three images of each samples were selected to determine signal intensity of phalloidin‐iFluor 488 and DAPI for each group. The GEL hydrogel sample was considered as 100% and used as a reference for comparison with MC/GEL hydrogels groups.

### 2.4 Statistical Analyses

The statistical analyses of the collected data in the present study were performed using GraphPad Prism 7 software (Boston, MA, USA). The results were analyzed by running a one‐way ANOVA followed by Dunnett's multiple comparisons test for paired comparisons. Average data extracted using from three independent experiments with *p* value threshold of < 0.05. The levels of significance in the graphs are indicated as follows: *p < 0.05, **p < 0.01, ***p < 0.001, and ****p < 0.0001.

## 3 Result and Discussion

### 3.1 FTIR Analysis

**FIGURE 1:**
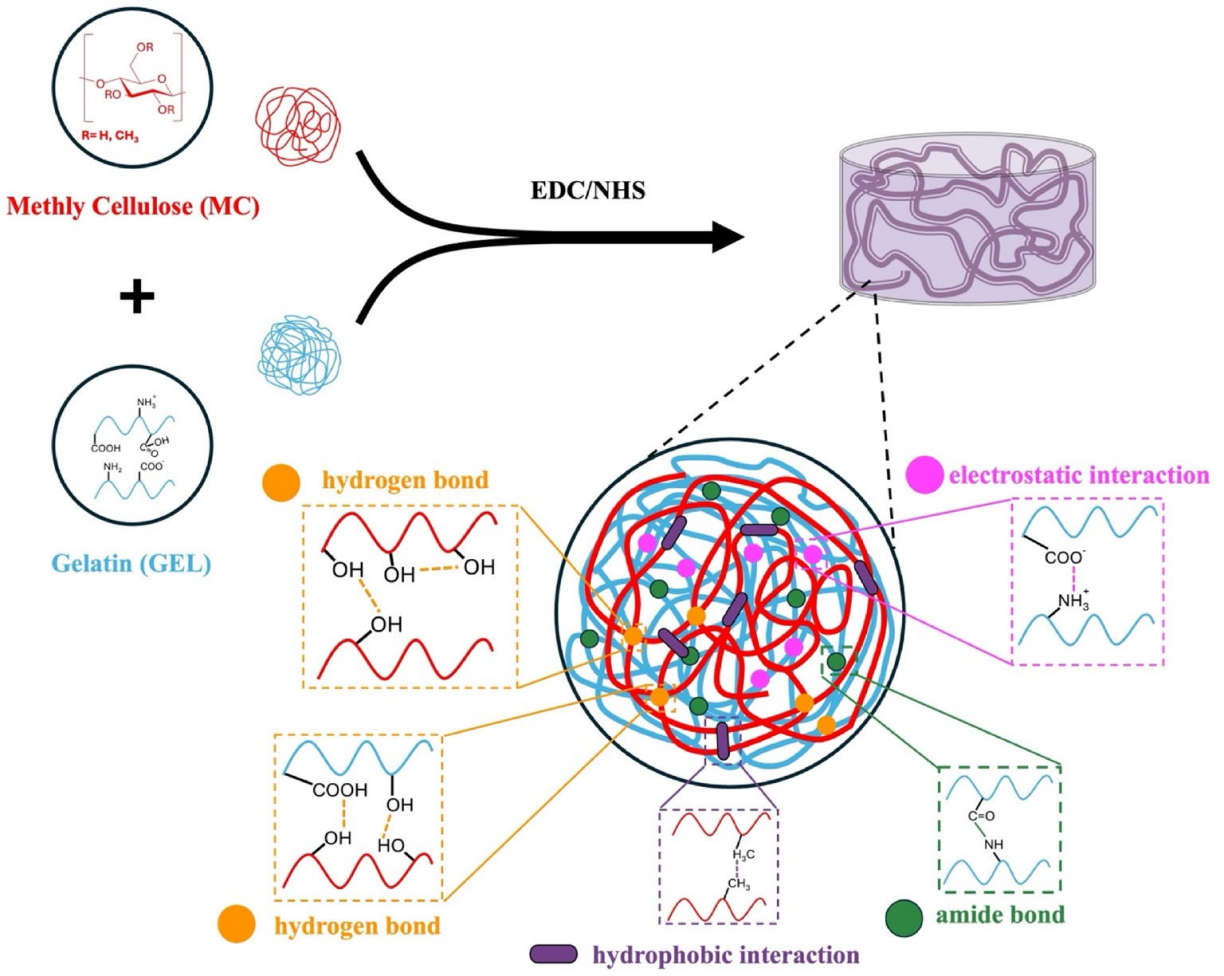
Physical and chemical interaction within the MC/GEL hydrogel.

In this study, MC/GEL‐based scaffolds were developed for cartilage tissue regeneration and evaluated for their physical properties and biocompatibility. Figure 1 schematically illustrates the intermolecular interaction between MC/GEL polymer chains.

**FIGURE 2:**
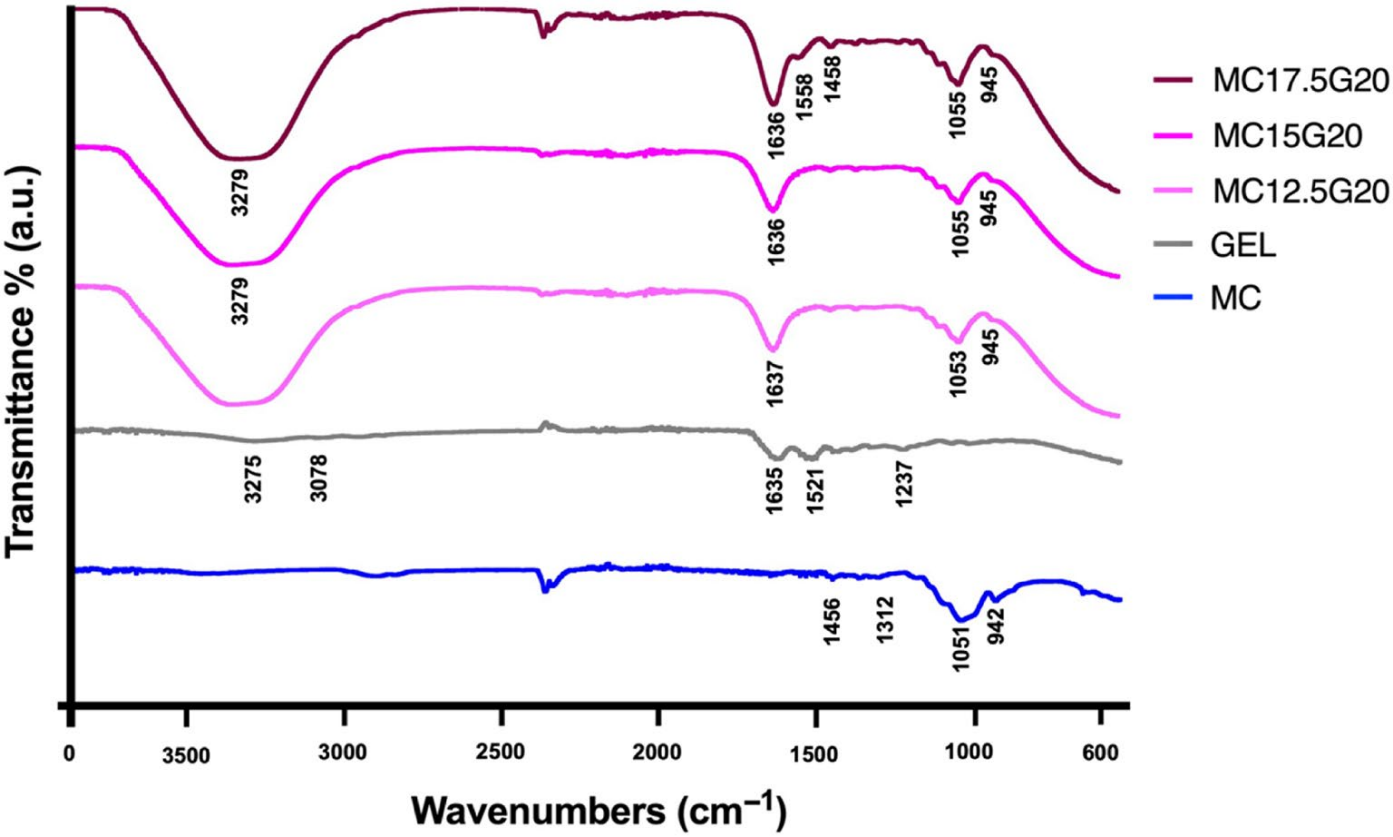
FTIR spectrum of GEL, MC, MC12.5G20, MC15G20, and MC17.5G20.

First, this figure illustrates the formation of amide bonds through the conjugation of COOH and NH_2_ group of GEL with EDC/NHS [23]. Second, the physical crosslinking through hydrogen bonding between MC and GEL chains is shown [24]. Additionally, the chain entanglement of polymer chains in MC/GEL contributes to the hydrogel's stability. Furthermore, MC chains exhibit hydrophobic interactions due to hydrophobic methyl groups [25]. Figure 2 presents the FTIR spectra of MC, GEL, MC12.5G20, MC15G20, and MC17.5G20.

FTIR analysis was performed to evaluate the interaction between MC and GEL after blending and subsequent crosslinking with 10 mM EDC and 50 mM NHS. The FTIR spectrum of GEL revealed five characteristic polypeptide bands: amide A, amide B, amide I, amide II, and amide III bands, located at 3275, 3078, 1635, 1521, and 1237 cm^−1^, respectively. The band at 3275 cm^−1^ (amide A) corresponds to the stretching vibration of NH group coupled with hydrogen bonding. Amide B, at 3078 cm^−1^, is attributed to the asymmetric stretching vibration of the C=H. The amide I band, observed at 1635 cm^−1^, was associated with the N—H bending vibration from GEL. The amide II band, at 1521 cm^−1^, represents N—H bending and C—N stretching vibrations. Finally, the amide band, around the 1237 cm^−1^, was attributed to N—H deformation [26, 27].

In the MC spectrum, the band at 1051 cm^−1^ was assigned to C—O—C stretching from glycosidic units of MC [28, 29]. The characteristic bands of MC at 1456 cm^−1^ represent in‐plane C—H deformation vibrations, while bands at 1373 and 942 cm^−1^ were attributed to the stretching of CH_2_ and CH_3_ groups, respectively.

After blending of GEL with MC, new absorption band at 3279 cm^−1^ was observed in the MC/GEL spectrum. This peak, attributed to the overlap of —OH bonds from MC and —NH groups from GEL which indicated the formation of hydrogen bonds. Additionally, the band at 1636 cm^−1^ corresponds to the COOH group of GEL, signifying electrostatic interaction between the COO^−^ ions of GEL and NH_3_^+^ ions of MC [30].

### 3.2 Mechanical Analysis

**FIGURE 3:**
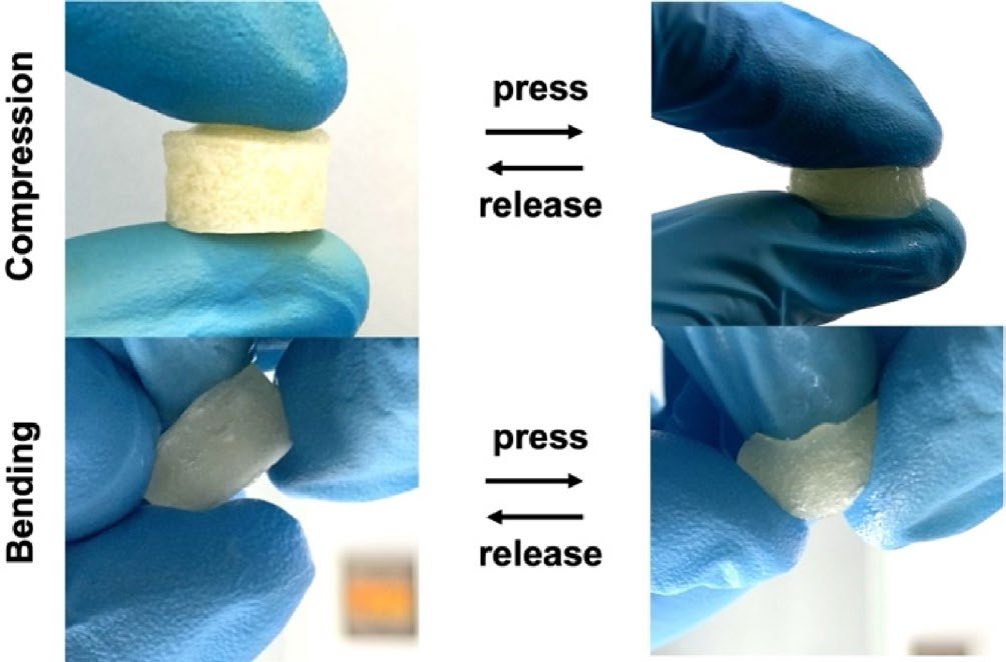
Images of the MC12.5Gel20 hydrogel under bending and compression conditions.

Figure 3 illustrates that the hydrogels exhibited high resistance to deformation and maintained significant flexibility during bending. The dynamic and reversible nature of hydrogen bonds contributes to this flexibility, allowing the MC/GEL hydrogel to rapid recovery to its original shape. This swift recovery is attributed to the abundant hydrogen bonding within the hydrogel structure [24, 31].

**FIGURE 4:**
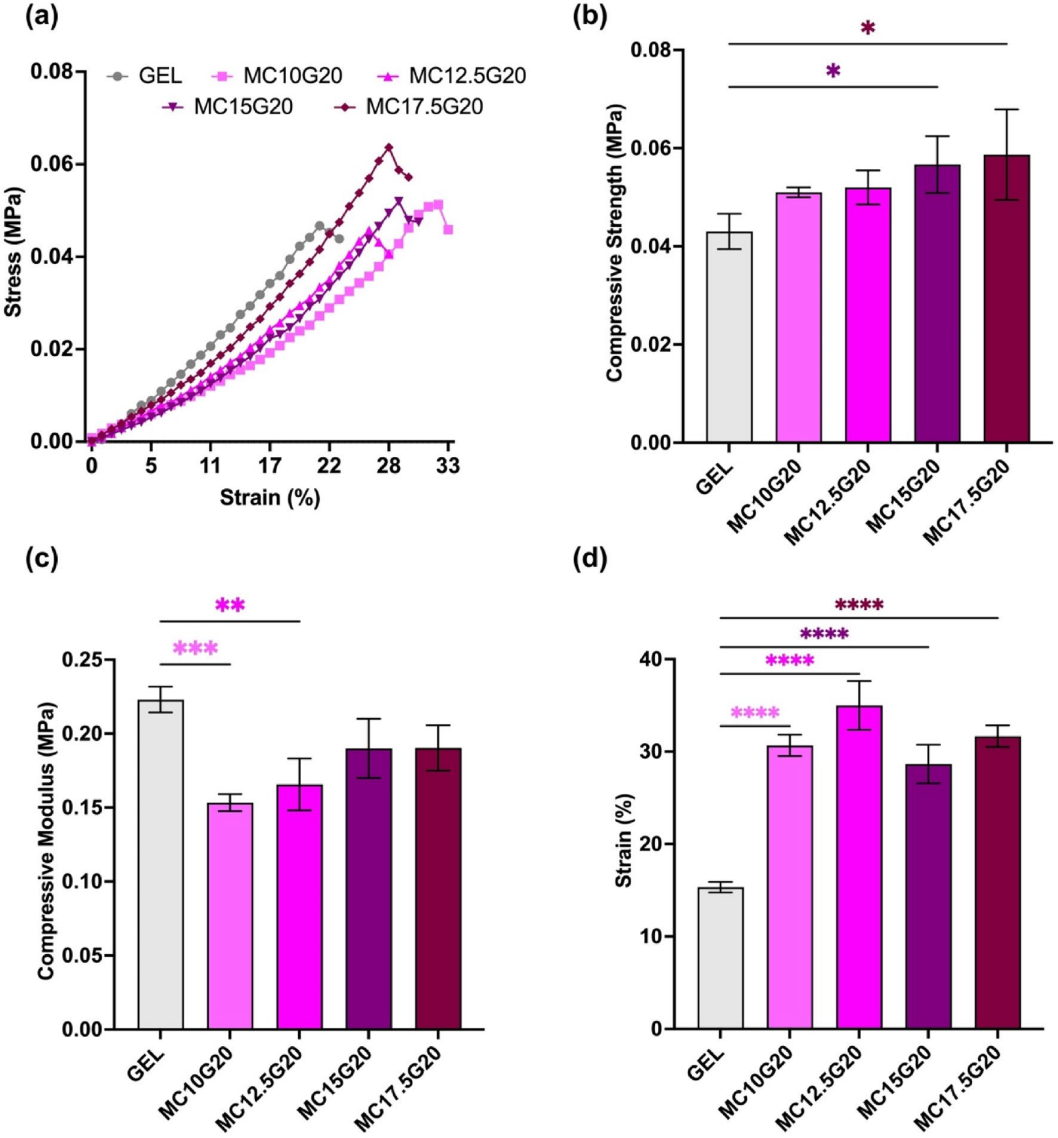
Mechanical performance of the hydrogels. (a) Stress–strain curve of hydrogel, (b) compressive strength curve, (c) compressive modulus curves, and (d) percentage strain at failure (*p < 0.05, **p < 0.01, ***p < 0.001, and ****p < 0.0001 compared with GEL.)

The mechanical properties of hydrogels influence the cellular responses of BM‐MSC cultured on them [32]. Therefore, in this study, the goal was to achieve cartilage‐like mechanical properties [32]. Figure 4 presents the mechanical characterization of hydrogels under compression.

The compression stress–strain curves shown in Figure 4a confirm that MC/GEL hydrogels exhibit a high percentage strain at break, indicating excellent ductility. As previously mentioned, this behavior is attributed to the presence of abundant reversible hydrogen bonds [31]. It was observed that as additional loading was applied, the gradient of the curves increased until the ultimate compressive strength was reached, which indicated strain hardening [33]. This effect became more pronounced with an increasing wt.% of MC in the hydrogels, demonstrating enhanced resilience as wt.% of MC increased [24]. Figure 4b shows that compressive strength improves with higher wt.% of MC indicating increased resistance for plastic deformation. This enhancement is likely due to chain entanglement between the MC and GEL polymer chains, as well as the presence of hydrogen bonding [34].

The compressive modulus of healthy articular human cartilage ranges from 0.24 to 0.85 MPa [35, 36]. In contrast, the compressive modulus of designed constructs for cartilage tissue engineering has been found to range from 0.005 to 5.9 MPa [37, 38]. In Figure 4c, the compressive modulus of the MC/GEL hydrogels reaches 0.20 MPa, which is close to the compressive modulus range of human cartilage tissue. However, as wt.% of MC increased, the compressive modulus decreased. This decline may be attributed to the lower compressive modulus of MC compared with GEL and disruption of amide bonds between GEL chains in the presence of MC chains [39]. Figure 4d confirms that MC/GEL hydrogels provide 2‐fold higher % strain at break compared with GEL.

### 3.3 Swelling‐pH Measurement of Hydrogels

**FIGURE 5:**
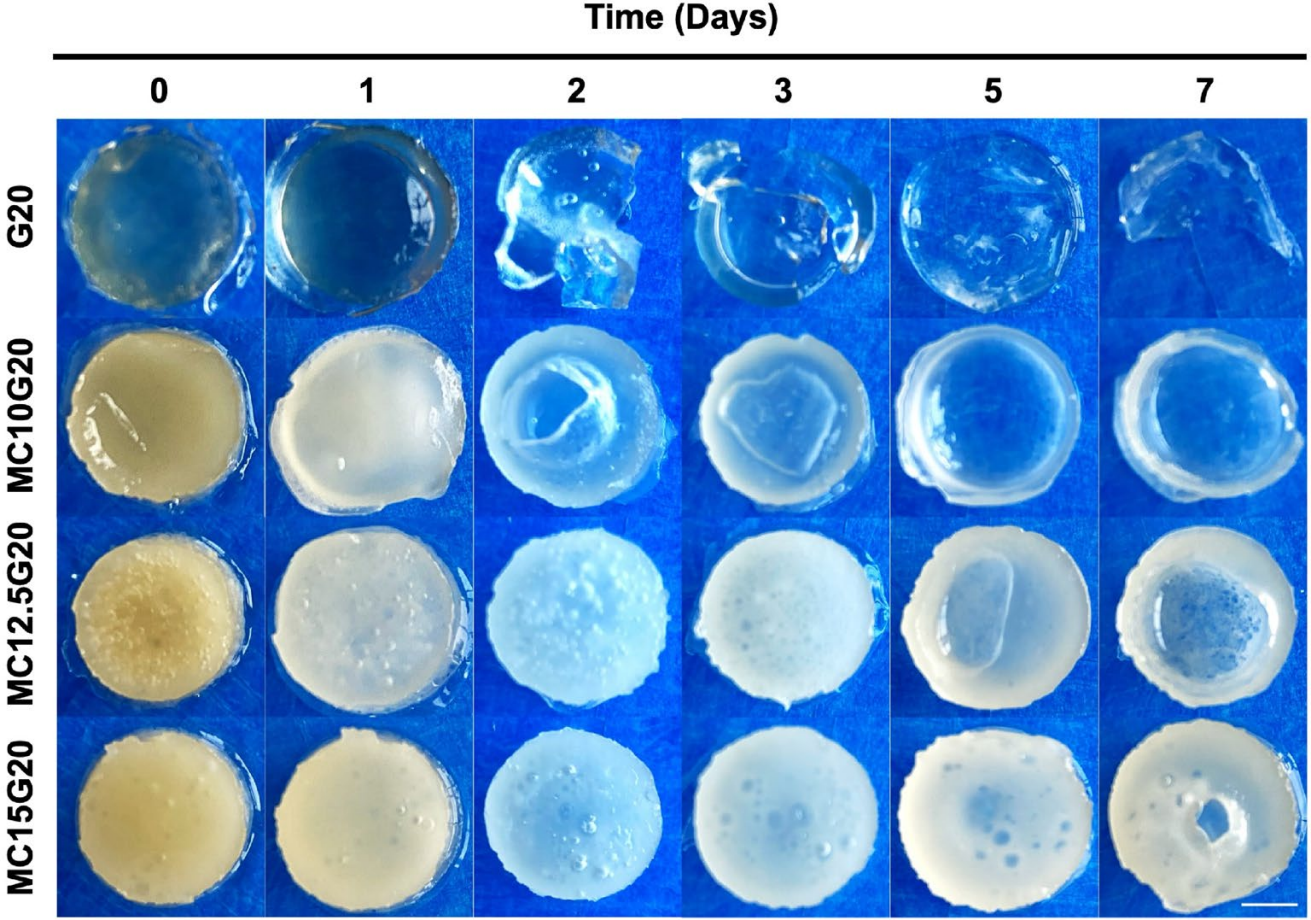
Digital photos of hydrogel tablets taken at six different time points (scale bar: 5 mm).

Swelling behavior and pH values of hydrogels were investigated for 7 days of incubation period in PBS solution at 37°C. As can be seen from Figure 5, hydrogels maintain preserve their structural integrity during incubation period when wt.% of MC increased.

**FIGURE 6:**
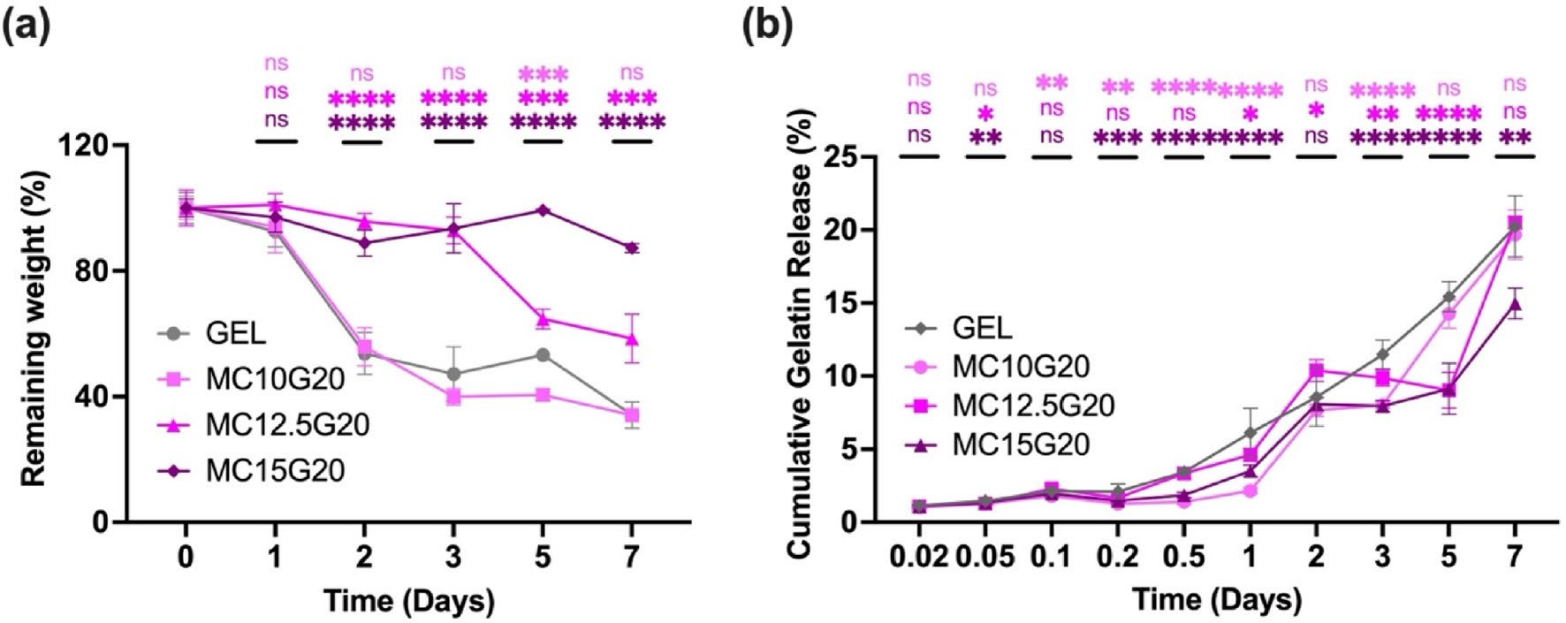
(a) % Remaining weight of hydrogels at given time points and (b) cumulative GEL release from MC/GEL hydrogel at given time points (the level of significance on the graphs are indicated as ns: not significant, *p < 0.05, **p < 0.01, ***p < 0.001, and ****p < 0.0001, in comparison with GEL).

Additionally, the water retention capacity within the hydrogel samples was enhanced by addition of MC. As shown in Figure 5, G20 began to degrade after Day 1. In contrast, with an increased MC ratio, degradation was significantly reduced; hydrogels with higher MC loading, maintained their integrity until Day 7. This enhanced stability is likely attributed to hydrogen bonding, chain entanglement, and hydrophobic interactions from methyl groups of MC [31, 40]. However, for MC/GEL groups, the degradation started from the inner layers, indicating bulk degradation of the hydrogels, a behavior also observed for bacterial cellulose (BC)‐based hydrogels [41]. Figure 6a illustrates the degradation profiles of the hydrogels, while Figure 6b represents the cumulative release of GEL over 7 days.

**FIGURE 7:**
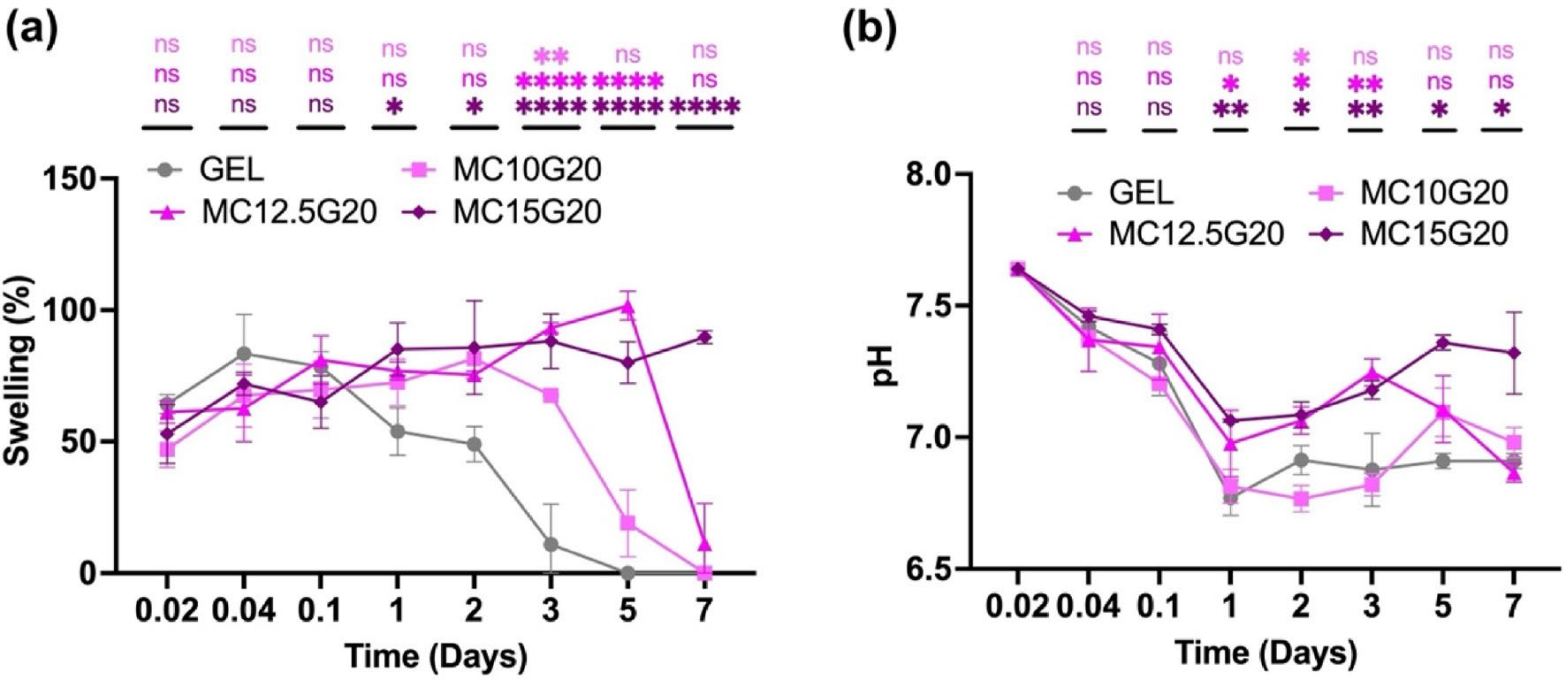
(a) Swelling ratio of hydrogel tablets and (b) pH changes in culture PBS of hydrogel tablets. The levels of significance are indicated as ns: not significant, *p < 0.05, **p < 0.01, and ****p < 0.0001 in comparison with GEL.

According to Figure 6a, GEL exhibited the fastest degradation among the hydrogels. The degradation rate of MC/GEL decreased with increasing MC concentration; for instance, MC15GEL20 preserved over 80% of its weight, while the remaining weight of GEL was around 40% after 7 days. Figure 6b illustrates the sustained release of GEL, which may might be correlated with stability of the hydrogels [42]. The diffusion of GEL from MC/GEL shows that a higher crosslinking density between MC and GEL provides greater resistance to the release of GEL. Our findings support previous studies showing that the release rate of GEL increases as crosslinking density between polysaccharides and proteins decreases [21, 43]. Furthermore, Figure 7 presents swelling ratio and pH of the hydrogels.

According to Figure 7a, all samples initially exhibited a swelling ratio of 50%, but MC15G20 retained this swelling until Day 7, indicating the structural integrity of MC15G20. This suggests that MC is beneficial in maintaining the form of GEL‐based hydrogels. Additionally, the variance in pH values of PBS solution from each hydrogel group were analyzed at each time point of the experiment. The pH values of PBS solution from each hydrogel group was analyzed at each time point during the experiment. The pH values of PBS solution remained within the range of human physiological pH. In this study, the decrease in pH of PBS solutions was more pronounced in GEL compared withMC12.5G20 and MC15G20 from Day 1 to Day 5. Physical stability and structural integrity of MC/GEL samples in PBS reduced ion dissolution, resulting in a relatively higher pH.

### 3.4 Morphology of Hydrogels

**FIGURE 8:**
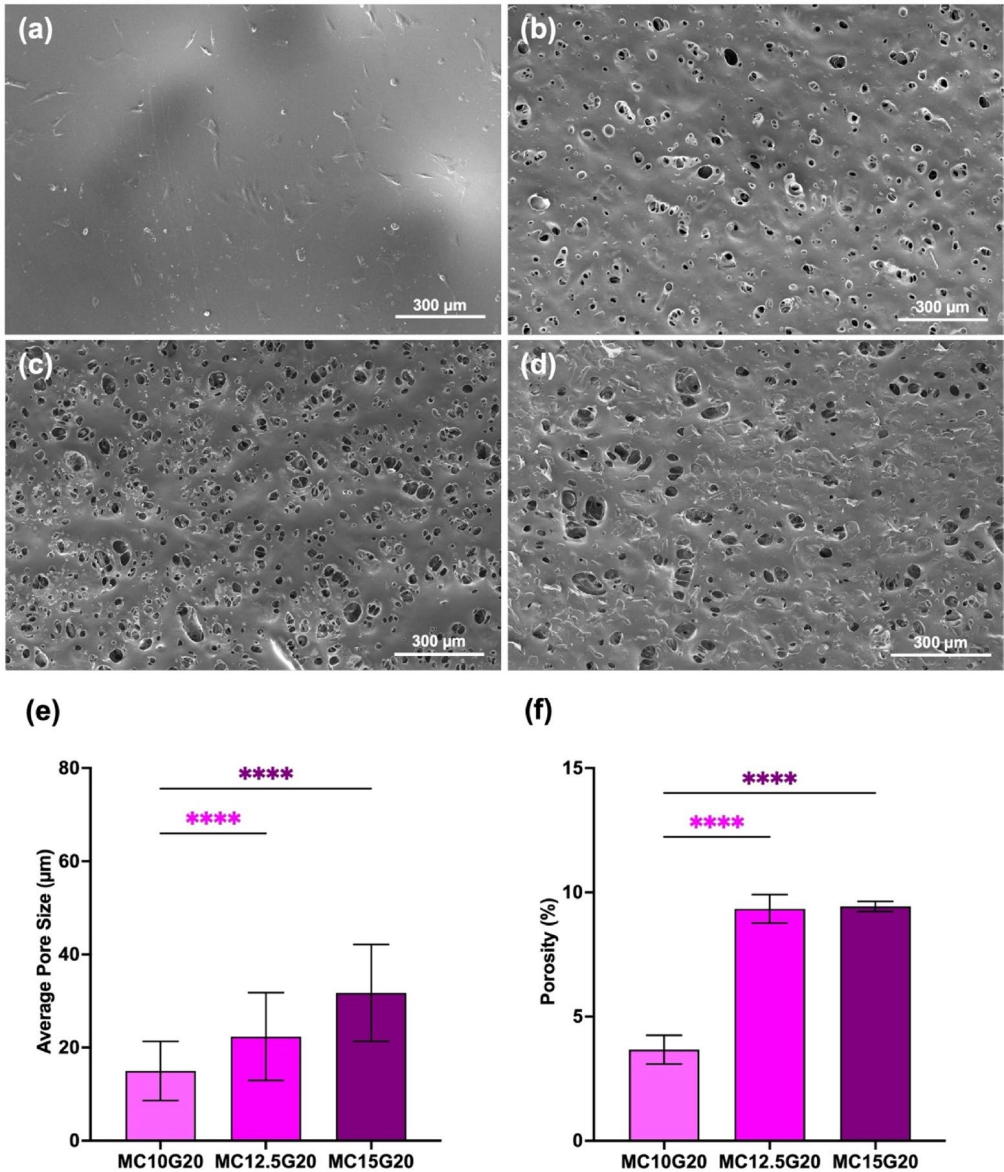
Scanning electron microscope images of (a) GEL, (b) MC10G20, (c) MC12.5G20, and (d) MC15G20. (e) Average pore size of MC/GEL hydrogels. (f) % Porosity of MC/GEL hydrogels (****p < 0.0001).

Porous network structure with interconnected porosity allows oxygen and nutrient transfer to support cell survival and function within hydrogels, promoting cell adhesion, proliferation of encapsulated cells, and the formation of tissue bridges across damaged tissue [44, 45]. After 5 days of the cell seeding on the hydrogel, morphological characteristics of hydrogel samples with varying MC content were compared with assess structural changes post‐cell culture. SEM images reveal that pure GEL hydrogels lack surface pores in Figure 8a. In contrast, however, hydrogels with added MC particles exhibit a porous structure, as shown in Figure 8b–d. The MC10G20 hydrogel demonstrated an average pore size of 15 μm, while MC12.5G20 showed pores ranging from 10 to 50 μm, similar to the pore size in cartilage tissue [46]. The MC15G20 with highest MC content, exhibited has larger pores compared with MC12.5G20 and MC10G20, as seen in Figure 8e. Previous studies confirm a 3D BC hydrogel network with interconnected pores, where porosity decreased after addition of gelatin [47]. On the other hand, it was found that the pore density increased up to 9.6% with increasing the MC concentration (up to MC12.5G20) which is shown in Figure 8f.

In Figure 8b–d, cell attachment is challenging to visualize due to the opacity of MC in contrast to the visible cell adhesion on the pure GEL hydrogel in Figure 8a. Cell attachment to the porous MC/GEL surface, shown at 2000× magnification in Figure S1, indicates that while MC‐added scaffolds support cell adhesion, it is less pronounced compared with pure GEL scaffolds. This reduced cell adhesion to the MC/GEL surface may be linked to the hydrophobicity of MC and fewer cell adhesion molecules in MC/GEL hydrogels compared with pure GEL hydrogels. To mitigate MC's negative impact on cell adhesion, future studies may investigate effect of reducing MC content in MC/GEL hydrogels.

### 3.5 Effect of Hydrogels on Metabolic Activity and Morphology of BM‐MSC

**FIGURE 9:**
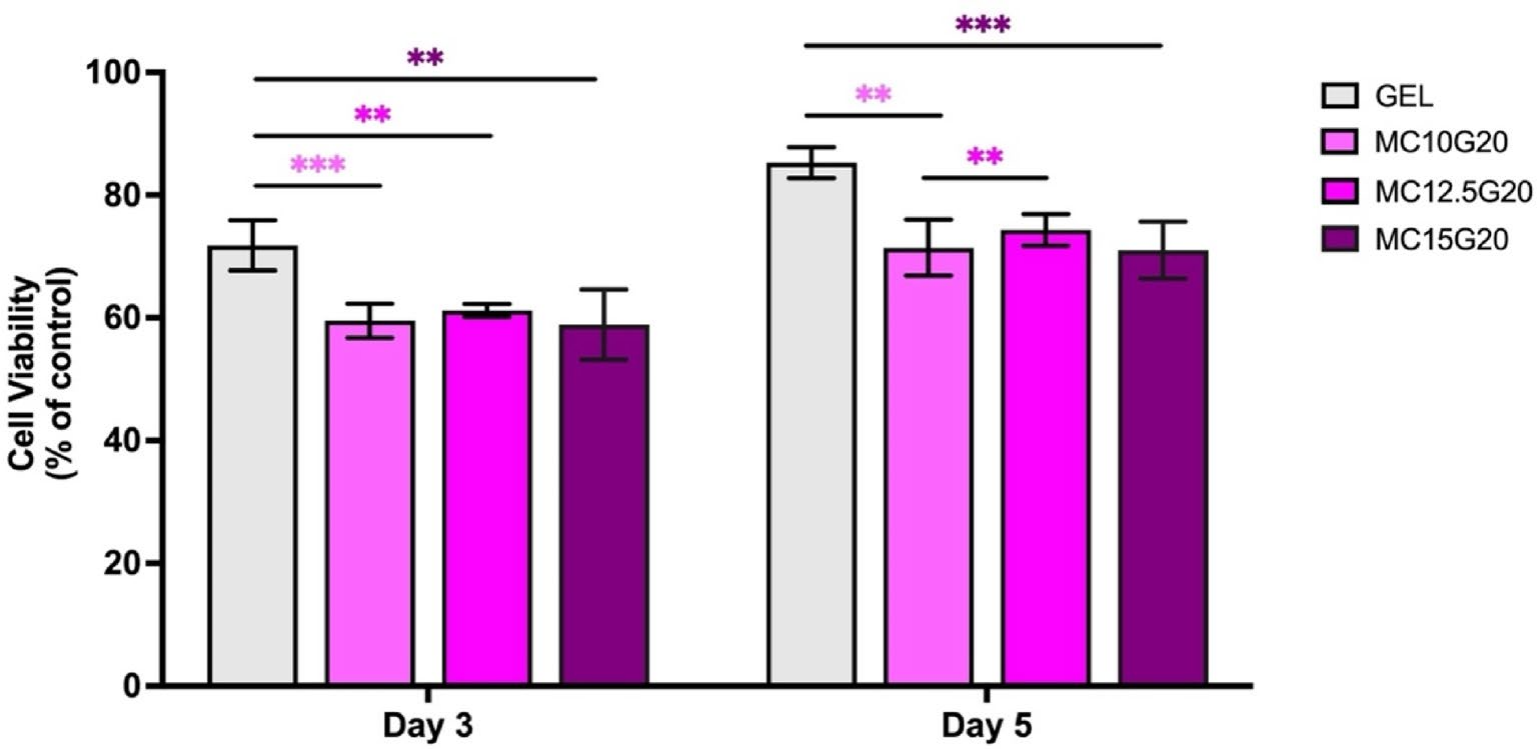
% Cell viability of BM‐MSCs cultured on the prepared hydrogels at days 3 and 5 (*p < 0.05, **p < 0.01, ***p < 0.001, and ****p < 0.0001).

Figure 9 shows relative % cell viability of BM‐MSC following direct cell culture test, conducted to assess the biological performance of MC/GEL hydrogels.

**FIGURE 10:**
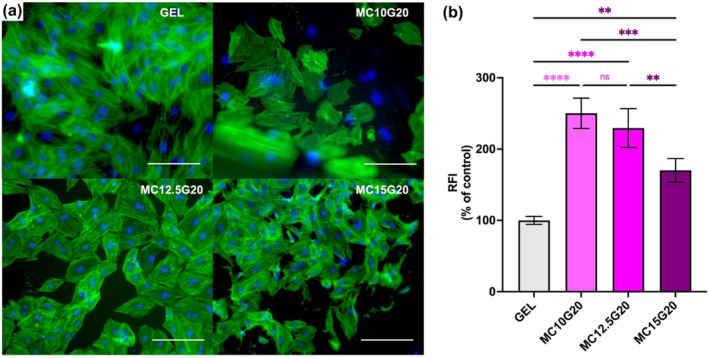
(a) Fluorescence images of BM‐MSC cells on Day 5 was shown (scale bar: 20 μm) and (b) quantitative analysis of relatively phalloidin‐iFluor intensity for BM‐MSCs (ns: not significant, **p < 0.01, ***p < 0.001, and ****p < 0.0001).

First, all study groups showed proliferation as % cell viability increased from Day 3 to Day 5. This result aligns with previous report showing higher % cell viability in GEL compared with BC and also higher % cell viability on Day 5 relative to Day 3 [48, 49]. The relatively lower % cell viability in MC hydrogels may be attributed to the limited adhesion molecules within MC hydrogels, reducing cell affinity for the hydrogel, and resulting in decreased % cell viability. Additionally, the hydrophobic nature of MC due to its hydrophobic groups may further hinder cell attachment to MC surface [50]. Nevertheless, 70% cell viability was achieved on Day 5 in MC incorporated groups, indicating the biocompatibility of all the hydrogels [51]. Figure 10 shows fluorescence images of sample revealing the spindle‐shaped morphology characteristic of BM‐MSC in each group.

Moreover, an elongated cell shape was observed in BM‐MSC cultured on GEL. In contrast, cells exhibited less elongation on MC/GEL compared with GEL and BM‐MSC on GEL had larger surface area than those on MC/GEL. The effect of GEL on cell elongation is supported by previous research, which shows that RGD peptide sequence in GEL provides better 3D scaffold for cell adhesion to rough surfaces [49, 52]. Although, cytoplasmic elongation and multicellular network of BM‐MSC on GEL were noted, quantitative analyses of the Figure 10b indicate that cells cultured on MC/GEL have a higher % F‐actin intensity/nucleus intensity compared with those on GEL [53]. Leipzig et al. [54] indicate that increased F‐actin intensity in chondrocytes following TGF‐b1 and IGF‐I exposure is associated with cellular stiffness. It can be inferred from Figure 10b that low concentration of MC enhance F‐actin intensity, potentially increasing cell stiffness and promoting MSC differentiation investigating the mechanism behind the significant increase in F‐actin intensity with incorporation of MC in the hydrogels would be worthwhile.

In the future, it would be beneficial to conduct antibacterial studies on the hydrogels to fully understand their potential for cartilage regeneration applications. This study also indicates that the stability of the samples requires further enhancement, as degradation was observed in the MC/GEL groups after 7 days. Furthermore, % cell viability decreased following the introduction of MC to GEL hydrogels, although biocompatibility was still exhibited biocompatibility. Therefore, the stability and in vitro cell culture behavior of the hydrogels may be further improved by incorporating carbon nanostructures such as graphene oxide or fullerenol. Additionally, cartilage repair experiments in mouse model are needed to demonstrate positive impact of MC/GEL hydrogels on cartilage formation. Overall, these findings confirm that MC/GEL hydrogel has significant potential for treating cartilage, but this should be supported by future in vitro and in vivo studies.

## 4 Conclusion

This study investigates the effect of MC concentration on the mechanical, chemical, and in vitro properties of MC/GEL hydrogels. FTIR analysis confirmed between MC and GEL through hydrogen bonding, as indicated by the introduction of a strong —OH band. SEM analyses demonstrated that the incorporation of MC into GEL hydrogels resulted in increased porosity. The swelling experiment indicates that MC enhanced the water retention capacity of the hydrogels. Additionally, MC improved the mechanical properties and stability of the hydrogels. The metabolic and morphological characteristics of BM‐MSCs were not significantly affected by addition of MC to GEL and biocompatibility was maintained, as shown by the Alamar blue assay. F‐actin staining was also enhanced with addition of MC to the hydrogels, which requires further investigation. Overall, this study suggests that MC/GEL hydrogels potentially provide a suitable environment for cartilage tissue engineering.

## Author Contributions

All authors contributed to the conceptualization and design of the study. M.A.K conducted the material preparation and data collection. All authors contributed to the analysis of the results. M.A.K. and D.E. contributed to the preparation of the first draft of the manuscript. All authors commented on all drafts of the manuscript. The final version of the manuscript was read and approved by all of the authors.

## Ethics Statement

The authors have nothing to report.

## Conflicts of Interest

The authors declare no conflicts of interest.

## Supporting information


Data S1.

## Data Availability

The data that support the findings of this study are available from the corresponding author upon reasonable request.
