# Supplementary material for: Highly Flexible Methyl Cellulose/Gelatin Hydrogels for Potential Cartilage Tissue Engineering Applications
Source: Biopolymers. 2025 Jan 7;116(1):e23641. doi: 10.1002/bip.23641 (PMC11707504; doi:10.1002/bip.23641)
Supplement: Supplementary file 1 — Data S1. [file BIP-116-e23641-s001.docx]

**SUPPLEMENTARY**


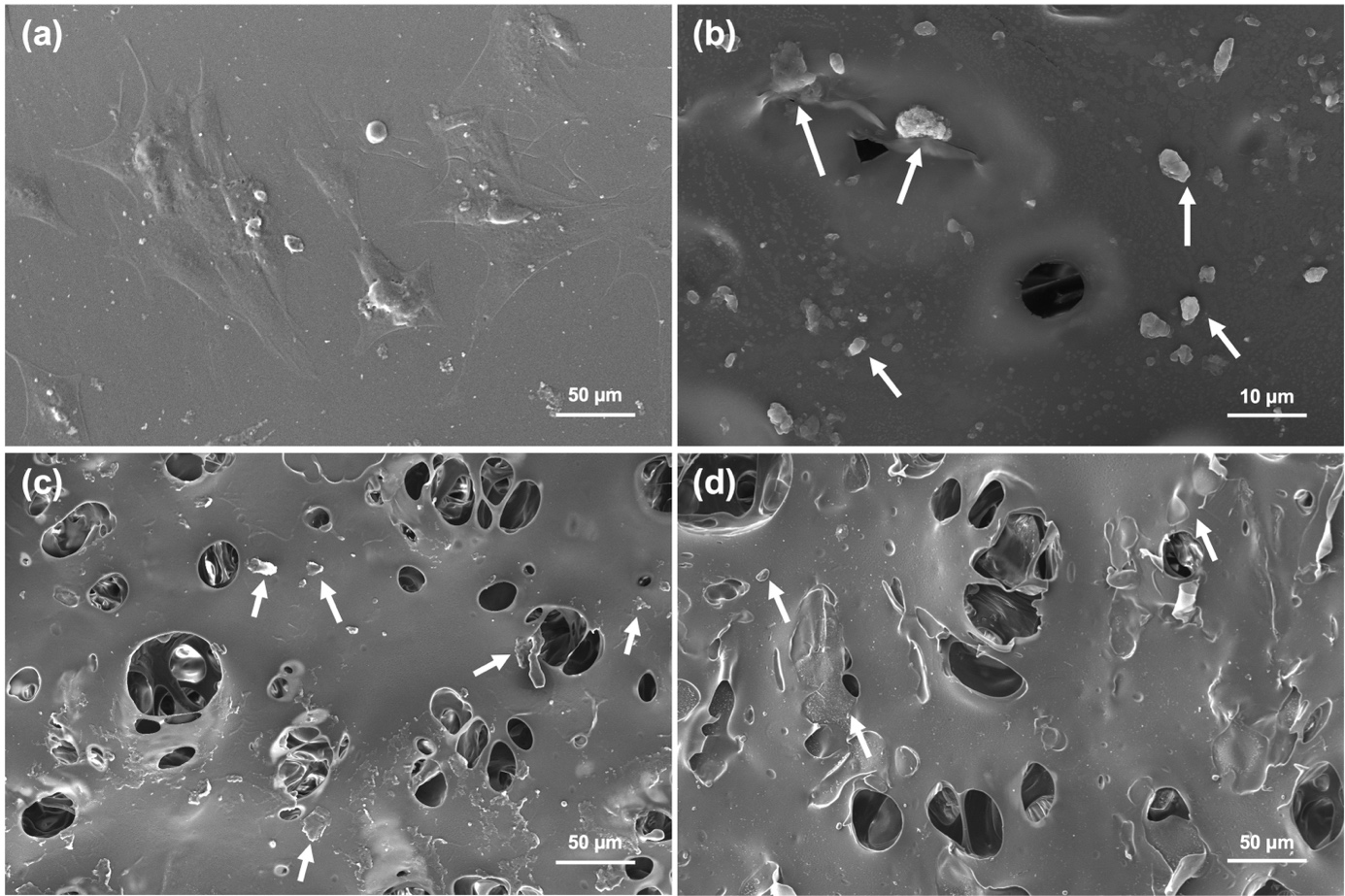


**FIGURE S1.** Scanning electron microscope images of (a) GEL, (b) MC10G20, (c) MC12.5G20, (d) MC15G20: BM-MSCs cells (arrow, b, c, and d).
